# Assessing individual differences in attitudes towards touch in treatment settings: Introducing the touch & health scale

**DOI:** 10.1177/20551029221137008

**Published:** 2022-11-14

**Authors:** Aikaterini Vafeiadou, Natalie C Bowling, Claudia Hammond, Michael J Banissy

**Affiliations:** 1 Department of Psychology, Goldsmiths, University of London, London, UK; 2 School of Human Sciences, 4918 University of Greenwich, London, UK; 3 Department of Psychology, 1948 University of Sussex, Falmer, UK; 4 School of Psychological Science, 1980 University of Bristol, Bristol, UK

**Keywords:** Health psychology, well-being, attachment style, scale, diversity, touch attitudes

## Abstract

Individuals commonly receive touch in treatment settings, but there is limited research on how they perceive it. The current project sought to address this gap by: 1) developing the Touch & Health Scale (THS) - a novel instrument to measure attitudes to touch in treatment settings 2) assessing inter-individual differences in THS scores, and 3) examining the association between individuals’ THS scores and wellbeing. Data of a large U.K. adults sample (*N* > 12,000) were used. THS showed Cronbach’s α between 0.636 and 0.816 and significant correlations (*p* < 0.001) with day-to-day attitudes to touch. THS scores differed as a function of extraversion and avoidant attachment style. Participants with more positive attitudes to touch in treatment settings showed greater wellbeing. Overall, the study highlights the importance of a personalised approach to touch in treatment settings and provides a new scale that may act as a screening tool for this purpose.

## Introduction

Touch has a variety of functions in medical, health and beauty treatment settings, acting as an integral part of the therapeutic process. Interpersonal touch can be initiated by the professionals of a given setting or the patient. This can include acting as a common form of gesture for greeting or departure that can build rapport (Phelan, 2009), but also as means of diagnosis, treatment, and nursing care (Basiri et al., 2016; Berendsen, 2017; Karagozoglu and Kahve, 2013; Kelly et al., 2018; Phelan, 2009).

Certain forms of touch have also been linked with positive health outcomes. Affection Exchange Theory argues that we use affective touch in favour of our survival (Floyd et al., 2018). For instance, gentle touch has been found to lower anxiety, stress, depressive symptoms and improve sleep patterns (Weze et al., 2007) and hugging is reported to promote a better mood and life satisfaction (Packheiser et al., 2022). Affective touch is also used in treatment settings and links to improved patients’ health (e.g., reduced anxiety) and patient-therapist/carer communication (Fleischer et al., 2009; Kim and Buschmann, 1999; Williams, 2001). In addition, therapeutic touch, such as having a massage, is associated with reduced stress (Field, 2019). However, it is essential to note that an individual’s comfort with touch might influence its positive health effects. For example, nurses’ increased comfort with touch is associated with reduced emotional exhaustion in the workplace (Pedrazza, Minuzzo, et al., 2015).

While touch offers many benefits, tactile interactions must be implemented with care (Durana, 1998; O’Lynn et al., 2017; Singh and Leder, 2012; Wearn et al., 2020). Bodily touch can be misinterpreted, risking discomfort to the patient. For example, touch can be linked with power differences in interpersonal contexts that often place the patient in a disadvantaged position compared to the professional (Jones and Glover, 2014; Twigg et al., 2011). Further, touch in treatment settings delivered by the professionals can be perceived as erotically-intentioned and, by extension, contribute to the loss of the safe space that the patient needs (Alyn, 1988; Twigg et al., 2011; Wearn et al., 2020).

Prior research on how touch is perceived in treatment settings supports that there is inter-individual variability in response to touch. Individuals’ view vary according to their cultural background, age, gender, and neurodiversity (Kelly et al., 2018; Phelan, 2009). However, it is worth noting that these observations come from a small pool of research that is not always demographically diverse (e.g., Singh and Leder, 2012). For instance, there is not always a clear distinction of age groups (Pasco et al., 2004), and only a few studies consider cultural differences in healthcare settings (Lu et al., 2014; Williams et al., 2013).

Individual differences may also indicate which forms of touch are comfortable for certain individuals (Pedrazza et al., 2018). There are different forms of touch in treatment settings (e.g., therapeutic, diagnostic) and an ongoing effort to categorise them according to their goal and effects from professionals’ and patients’ points of view (Davin et al., 2019; De Luca et al., 2021; Karlsson et al., 2022; Kelly et al., 2018; Pedrazza et al., 2018; Pedrazza, Trifiletti, et al., 2015). For instance, female nurses are reported to feel more comfortable engaging in touch that promotes emotional containment than male nurses (Pedrazza et al., 2018). However, the touch terminology of a given setting does not always appear consistent in the literature (Gleeson and Timmins, 2004). With this in mind, further investigation into how individual differences affect attitudes towards the different forms of touch in treatment settings is needed.

There are some individual differences that are known to exert a wider impact on general day-to-day attitudes towards touch. For example, culture is a defining factor of touch frequency and appreciation (Dibiase and Gunnoe, 2004). In Western cultures, pleasantness ratings of interpersonal touch are higher than in East Asian cultures (Katsumi et al., 2017; Suvilehto et al., 2019). Similarly, daily tactile gestures, such as a greeting handshake, are more frequent in Western than in East Asian cultures (Katsumi et al., 2017; Suvilehto et al., 2019). Apart from culture, women are sometimes reported to have more positive attitudes to touch than men (Pedrazza et al., 2018; Trotter et al., 2018; Webb and Peck, 2015). Another important aspect of touch attitudes is the age of the individual, although findings are mixed. Some suggest that the pleasantness of touch increases during ageing (Sehlstedt et al., 2016; Webb and Peck, 2015). However, the latter age effect was not observed by Trotter et al. (2018) (please note that in this study, the majority of their participants were below 30 years old, so outcomes require further investigation). While these factors (culture, gender and age) have been examined in wider interpersonal contexts, their impact on individual differences in attitudes towards touch in treatment remains under-investigated.

There are also psychological traits that could have an impact on touch attitudes in treatment settings, but remain understudied. Previous research has shown that the ‘Big Five’ personality traits extraversion, agreeableness, conscientiousness, and openness predict positive attitudes toward massage treatments, while neuroticism is a negative predictor (Moyer and Rounds, 2009). Agreeableness is described as a positive predictor of touch in intimate body parts (i.e. chest, thigh and buttocks) and non-intimate body regions, while openness was a positive predictor only for touch attitudes towards non-intimate regions (Dorros et al., 2008).

In addition to personality traits, attachment style is also a key factor of interpersonal touch. Attachment style refers to how individuals relate in close relationships based on their infant-primary-caregiver past experiences (Bowlby, 1969). During development, infants seek comfort, support, and security from their caregivers, and touch plays a critical role in communicating these needs. For example, the infant may cling to their caregiver to show their need for physical contact and fear of separation (Cavalli, 2014; Field et al., 2005). Caregivers also use touch to validate and respond to infants’ needs, such as cuddling or gently stroking the infant’s back to provide calmness (Harrison, 2001; Van Puyvelde et al., 2021). The way and frequency of the caregiver’s response to infants’ needs, including the touch element of this interaction, may result in a different attachment style and comfort with intimate touch during adulthood (Beltrán et al., 2020).

Here two attachment styles have been linked to day-to-day tactile attitudes and experiences: avoidant-attachment style (fear of intimacy and preference for maintaining independence in relationships) and anxious attachment style (difficulty trusting others in relationships and worry about abandonment). People who score higher in avoidant attachment tend to be less tolerant to close interpersonal proximity, a requirement for receiving touch, compared to individuals with anxious attachment style (Kaitz et al., 2004). Individuals with anxious attachment seem to benefit more from the analgesic effects of pleasant touch when compared to avoidant individuals (Krahé et al., 2016; Von Mohr et al., 2018). Although a body of research investigates attachment style in relation to attitudes towards touch from a nurse’s point of view (Pedrazza et al., 2018) a patients’ perspective requires further investigation.

Prior work has shown that individuals with more positive feelings about their own body are also more positive about being touched, in everyday situations (Orbach and Mikulincer, 1998). In another study, craniofacial massage exerted a beneficial effect on the negative body image of menopausal women (Espí-López et al., 2020). However, these associations between touch and body image were reported for clinical samples, and there is little research investigating similar patterns in typical adults (Dunigan et al., 2011; Gupta et al., 1995).

Finally, pleasant touch is considered as an interoceptive signal because it shares a common neuronal pathway with the processing of other interoceptive modalities (e.g., Björnsdotter et al., 2010). Thus, it is also possible that increased interoceptive awareness could influence the perception of touch in treatment settings.

Although the findings above point to potential factors that may influence individual’s touch attitudes in treatment settings, few studies have systematically investigated this from a patient’s point of view (Tanzer et al., 2022). We sought to address this gap by developing a new measure (the ‘Touch & Health Scale’ - THS) that quantifies individuals’ touch attitudes in treatment settings. The scale sought to investigate three aspects of individuals’ touch in treatment settings: willingness to engage in tactile treatments, communicative behaviour when receiving touch in treatment settings, and feeling of comfort with receiving touch in medical settings. We employed the THS scale in a cross-sectional study among other measures, resulting in a large and diverse adult sample (>12,000 adults) of the U.K. general population. We used this large dataset to evaluate the psychometrics of THS and calculate the composite scores of the three TH Subscales. We then examined which individual differences may act as predictors of the three TH Subscales’ scores and whether these scores can act as predictors of individuals’ wellbeing. Specifically, we sought to address the following questions:

1: What are the psychometric properties of the THS, and how does the scale relate to scales of day-to-day attitudes towards touch in non-treatment settings? We predicted that more broad positive day-to-day attitudes to touch would relate to more positive attitudes to touch in treatment settings.
2: How do attitudes to touch in treatment settings change as a function of inter-individual differences in age, gender, personality traits, attachment style, interoceptive awareness, and body image? We predicted that older adults and females would show the most positive attitudes. We also predicted that personality (high scores in extraversion, agreeableness, conscientiousness and openness, low scores in neuroticism), attachment style (high anxious attachment scores, low avoidant attachment scores), higher body image acceptance, and higher perceived interoceptive ability would be associated with more positive attitudes to touch in treatment settings.
3: How do attitudes and experiences of touch in treatment settings relate to mental wellbeing and social wellbeing (hereinafter called “loneliness”)? We predicted that more positive attitudes towards touch in treatment settings would be linked with greater mental wellbeing and reduced loneliness.

Inter-personal touch in treatment settings can influence the professionals and patients involved. However, we would like to highlight that the above three questions focus only on attitudes and experiences from a patient’s point of view. Please also note that we use the word “patient” to describe individuals that seek any treatment that is not necessarily hospital-based or delivered by a medical professional (such as having a massage).

## Methods

### Procedure

Data for this study are drawn from The ‘Touch Test’, an online self-reported cross-sectional survey that explored attitudes to touch in a worldwide sample. The Touch Test was conducted between 20/01/2020 and 31/03/2020 and participants were recruited using opportunity sampling. Participants gave online informed consent, were required to be aged 18 or over and have internet access on a computer, smartphone or tablet to complete the survey. Participation was voluntary without receiving any monetary reward. After starting the survey, participants had 7 days to complete the study.

### Participants

Healthy adult UK participants are investigated as this was the region from which the largest number of participants were recruited. Healthy individuals were identified by reporting no current disability, long-term condition, or impairment. In addition, only individuals identifying as women or men who replied to all the Touch & Health Scale items, are included in the study. All sample selection criteria were pre-registered (Touch & Health Pre-Registration)^1^. This resulted in a sample of 12,291 healthy UK participants (9346 Females: 2945 Males, age: *M* = 56.57, *SD* = 13.60, age range: 18–92 years).

### Measures

The Touch Test contained a number of measures (please see the Touch Experiences & Attitudes Pre-Registration for a full list of measures). The measures were chosen according to their wide use in touch literature. In addition, shortened versions of a given measure were used to avoid increasing the length of the Touch Test study. For the current study, we investigated the following measures as per our pre-registered analysis plans:

#### The touch & health scale (THS)

This measure consisted of 14 items that measure attitudes towards touch in treatment settings (medical and non-medical). The THS items were measured on a five-point scale, with scores ranging from 1–5 for each item (strongly disagree to strongly agree respectively). Half of the items were reverse scored (see Table 1 for item details). Total scores (sum of responses) could range between 14–70, with higher scores indicating more positive attitudes to touch.

**Table 1.** The 14 items of the touch & health scale.

1. I feel that I can talk more openly to my doctor if they touch me.
2. I would feel more comfortable being touched by a machine than a person for a medical examination.*
3. I avoid tactile based treatments.*
4. Tactile based treatments make me feel calm.
5. I find tactile based treatments uncomfortable.*
6. I find that I can talk more to people while having tactile based treatments.
7. I would feel more comfortable being touched by a medical professional if they could not see me.*
8. I do not feel that medical professionals touch me often enough.
9. Touch from a professional helps to build my trust.
10. I regularly have massage treatments.
11. I would not like to be touched by a therapist, counsellor or psychologist. *
12. Touch from a therapist or counsellor psychologist would make me feel at ease.
13. I think it is inappropriate for a medical professional to hug a patient.*
14. I would prefer it if my doctor did not touch me.*

Questions with * are reversed scored items.

**Modified version of the Touch Experiences and Attitudes Questionnaire** (TEAQ; Trotter et al., 2018): This measure consisted of 12 items that represent six subscales related to: Childhood Touch (ChT), Friends and Family Touch (FFT), Current Intimate Touch (CIT), Attitude to Intimate Touch (AIT), Attitude to Self-Care (ASC), and Attitude to Unfamiliar Touch (AUT). The 12 items were selected by taking the two highest-loading items from each of the six published subscales on the original TEAQ (Trotter et al., 2018). Exploratory Factor Analyses of TEAQ data from the Touch Test survey indicated that the factor structure largely matched the six published subscales. However, two of the FFT items loaded also on ASC (Bowling et al., in preparation). To facilitate comparisons with prior published research, the TEAQ subscale scores were measured according to the published TEAQ version disregarding the small deviation in factor structure observed by the confirmatory factor analysis applied in our dataset (Bowling et al., in preparation). Therefore, each subscale scores range between 2–10, with higher scores indicating more positive attitudes to touch. The modified TEAQ is reported as a reliable instrument to measure attitudes to touch, with Cronbach’s α of the TEAQ subscales ranging from 0.54 to 0.89 (Cronbach’s α_*Attitude to Intimate Touch*_ = 0.75, α_*Attitude to Unfamiliar Touch*_ = 0.89, α_*Current Intimate Touch*_ = 0.81, α_*Childhood Touch*_ = 0.74, α_*Attitude to Self-Care*_ = 0.54, α_*Friends and Family Touch*_ = 0.62). The convergent validity of the modified TEAQ version was tested using the 17-item revised version of Social Touch Questionnaire (STQ) subscales and showed significant Pearson’s correlations with the majority of TEAQ subscales (*p* < 0.001) with varying magnitude (Pearson’s r = −0.712 to −0.036) (Bowling et al., in preparation).

**Social Touch Questionnaire** (STQ; Wilhelm et al., 2001): This measure consisted of 20 items that measure attitudes towards social touch, that represent three subscales related to: Dislike of Physical Touch (DPT), Liking of Familiar Physical Touch (LFPT) and Liking of Public Physical Touch (LPPT). Confirmatory Factor analyses of STQ data of the Touch Test survey indicated that the subscales largely matched the three published subscales (Bowling et al., in preparation). However, similar to TEAQ, the STQ published version is used for the validation of THS DPT scores range between 0–40, LFPT scores range between 0–24 and LPPT scores range between 0–16. For each subscale, lower scores indicate more positive attitudes to touch. STQ is reported as a reliable instrument to measure attitudes to touch, with Cronbach’s α of the STQ subscales ranging from 0.57 to 0.82 as reported by Vieira et al. (2016) (α_*Dislike of Physical Touch*_ = 0.68, α_*Liking of Public Physical Touch*_ = 0.75, α_*Liking of Familiar Physical Touch*_ = 0.71) and by (Bowling et al., in preparation) (α_*Dislike of Physical Touc*h_ = 0.82, α_*Liking of Public Physical Touch*_ = 0.82, α_*Liking of Familiar Physical Touch*_ = 0.57). The convergent validity of the STQ subscales was tested with the Social Interaction and Performance Anxiety and Avoidance scales showing significant correlations (*p* < 0.0001) with Pearson’s r > 0.5 (Vieira et al., 2016).

#### Mental Wellbeing and Loneliness

These constructs were measured by the Short Warwick–Edinburgh Mental Well-Being Scale (SWEMWBS; Stewart-Brown et al., 2009) and the revised UCLA Loneliness Scale (Russell et al., 1980), respectively. Mental wellbeing scores range between 7–35, with higher scores indicating better wellbeing. Loneliness scores range between 20–80, with higher scores indicating an increased feeling of loneliness. The 7-item SWEMWBS is reported as a reliable instrument to measure mental wellbeing with strict unidimensionality according to the Rasch model analysis of fit and validity tested by correlating it with the original 14-item WEMWBS (Spearman’s rho = 0.95) (Stewart-Brown et al., 2009). Similarly, the revised UCLA Loneliness scale is reported as a reliable instrument to measure loneliness with Cronbach’s *α* = 0.94 and validity tested by correlating it with the Beck Depression Inventory (r = 0.62), the Depression scale (r = 0.55) and the Costello-Comrey Anxiety scale (r = 0.32) (Russell et al., 1980). Additional supplemental analyses were run using the four items and eight items short version of UCLA (Hays and Dimatteo, 1987; Russell et al., 1980).

#### Attachment style

This was measured by the Experiences in Close Relationship Scale-Short Form (ECR-12; Lafontaine et al., 2015), which investigates two subtypes: Anxious and Avoidant attachment styles. Both subscales’ scores range between 6–42, with higher scores indicating higher anxious or avoidant traits. The scale is reported as a reliable instrument to measure attachment style, with Cronbach’s α values ranging from *α*_*Attachment Anxiety*_ = 0.87 to *α*_*Attachment Avoidance*_ = 0.79. The two Attachment subscales are also reported as significant predictors (*p*-values ranged from < 0.001 to < 0.05) of measures related to relationship satisfaction and psychological distress (Lafontaine et al., 2015).

#### Personality traits

Agreeableness, Conscientiousness, Extraversion, Openness to Experience, Neuroticism were measured by the short Big Five Inventory (BFI-S; Hahn et al., 2012). Each trait is represented by scores that range between 3–21, with higher values indicating a greater attribute of the given trait. The scale is reported as a reliable measure with Cronbach’s a values ranging between 0.44–76(*α*_*Agreeableness*_ = 0.44, *α*_*Conscientiousness*_ = 0.60, *α*_*Extraversion*_ = 0.76, *α*_*Openness*_ = 0.58, *α*_*Neuroticism*_ = 0.66). The scale’s convergent validity tested with the revised NEO-Personality Inventory showed significant correlations (*p* < 0.01) with average correlation coefficient = 0.60 (Hahn et al., 2012).

#### Interoceptive accuracy

The Interoceptive Accuracy Scale (IAS; Murphy et al., 2019) was used as a measure that assesses one’s self-reported interoceptive accuracy across a range of sensations. IAS scores range between 21–105. Higher scores relate to higher interoceptive accuracy. The scale is reported as a reliable measure of interoceptive accuracy with Cronbach’s *α* = 0.88 and convergent validity assessed with the Toronto Alexithymia Scale (Pearson’s r = −0.43, *p* < 0.001; Murphy et al., 2019). Additional analyses were run for the IAS version by excluding 5-items relevant to COVID-19 (breathing, cough, temperature, tired/sore muscles and taste).

#### Body acceptance

This was measured by the body acceptance subscale of the Dresden Body Image Questionnaire (DBIQ; Scheffers et al., 2017). The scores range between 5–25, with higher scores indicating greater body acceptance. The subscale is reported as a reliable measure of body acceptance by Scheffers et al. (2017), having a strong correlation (*r* = 0.96) with the original body acceptance subscale (Pöhlmann et al., 2013).

### Analysis

All analyses were pre-registered (Touch & Health Pre-Registration) and conducted in line with these plans. From the total group of 12,291 participants, subsequent groups are defined according to the variables of interest of each analysis. Subscales are treated individually, where a participant may be excluded from analysis for one subscale, but they can still be included in analyses for other subscales on that scale.

For the association of individual differences (mental wellbeing, loneliness, interoceptive accuracy and body acceptance scales and attachment style subscales), participants were included if they replied to at least 80% of the items of the scales/subscales of interest. The subscales of TEAQ, STQ and personality traits were included only when all the items of each subscale were present due to the small number of items per each subscale (please see Supplemental Materials Table 1 for sample size and demographic information of each scale/subscale).

The significance level for all correlation analyses was set to *p* ≤ 0.003; corrected for 18 correlations run in total for each THS subscale (please see Results section i for the description of THS subscales as identified by the exploratory factor analysis). The significance level for all the Mann Whitney comparisons was set to *p* ≤ 0.006; corrected for 8 comparisons run in total for each THS subscale. Finally, the significance level for all the regression analyses was set to *p* ≤ 0.01 for a given predictor.

### Data sharing statement

The current article includes the complete de-identified data-set used for its data analysis, which is a subset of the Touch Test raw data-set (available here: Touch Test Data). The SPSS data-set, SPSS codebook, SPSS syntax files, R code, the output of the SPSS syntax and R code files, the re-coded variables file and the explanatory memo (README.pdf) can be accessed here: Touch & Health Pre-Registration. Please use the README.pdf file as a guide to the different data files.

## Results

### What are the psychometric properties of the THS?

The factorability of the THS was assessed by Bartlett’s chi-square (χ^2^ > 0.30 minimum acceptable value) and Kaiser-Meyer-Olkin value (*KMO* > 0.60 minimum acceptable value). Exploratory factor analysis (EFA) was performed to identify common factors of the scale. Maximum Likelihood (ML) was used as method of factor extraction and direct oblique rotation was applied, since we expected correlation between the factors. The acceptable number of factors was assessed by Eigenvalues ≥ 1. In the factor loading process a minimum acceptable loading of 0.4 was applied.

The reliability of the THS was assessed by Cronbach’s alpha statistic (≥ 0.60 minimum acceptable value), as a measure of internal consistency of the TH items and its subscales indicated by the EFA. Our Cronbach’s α = 0.60 cut-off decision was made considering two observations: i) while Cronbach’s cut-off of α = 0.70 is commonly reported as the minimum acceptable value (Durani et al., 2009; Taber, 2018; Tavakol and Dennick, 2011), Cronbach’s α value of a scale can be influenced by the number of its items (Ponterotto and Ruckdeschel, 2007; Vaske et al., 2017). Small number of items may lead to low Cronbach’s α values (Vaske et al., 2017) and specifically for scales/subscales that have *n* < 6 number of items, Cronbach’s α = 0.60 is described as “fair” (Ponterotto and Ruckdeschel, 2007) ii) Cronbach’s α = 0.60 is characterised acceptable by some literature (Ponterotto and Ruckdeschel, 2007; Taber, 2018; Van Griethuijsen et al., 2015). Considering Cronbach’s α susceptible nature to the number of items, literature also suggests that supplement measures could be reported (Agbo, 2010). Thus, to better understand THS’s internal consistency, we also calculated the average inter-item correlational analysis as supplemental information.

Assessment of the convergent validity of the THS was performed via Pearson’s bootstrapped correlation analyses (1000 samples) between the THS subscales (factors indicated by EFA) and the STQ and TEAQ subscales.

#### Factor analysis

Kaiser-Meyer-Olkin value (*KMO* = 0.901) and Barlett’s Test (χ^2^ = 63167.32, *p* < 0.001) verified factorability of the data. The EFA showed that 57.9 % of variance can be explained by 3 underlying factors; Factor 1: Engagement in Tactile Treatments, Factor 2: Communication Facilitation via Touch, and Factor 3: Comfort with Touch in Medical settings (see Table 2). The 11^th^ and 13^th^ items had low-level loadings (< 0.40; see Table 2) and thus, they were disregarded when computing the total scores of each factor. The strongest correlation between the factors was observed between Engagement in Tactile Treatments and Comfort with Touch in Medical settings (see Supplemental Materials Table 2).

**Table 2.** Pattern matrix element loadings and the communalities of the exploratory factor analysis of the 14 items of the touch & health scale. The items that are high level loadings for a given factor are depicted in bold.

Item	Factor 1	Factor 2	Factor 3	h
TH_1	−0.063	**0.788**	−0.019	0.570
TH_2	0.120	0.030	**0.483**	0.307
TH_3	**0.705**	−0.042	0.277	0.686
TH_4	**0.691**	0.194	−0.005	0.632
TH_5	**0.709**	−0.005	0.275	0.720
TH_6	0.227	**0.522**	−0.087	0.389
TH_7	0.042	−0.110	**0.552**	0.285
TH_8	−0.003	**0.598**	−0.026	0.345
TH_9	0.085	**0.677**	0.048	0.548
TH_10	**0.597**	0.021	−0.113	0.328
TH_11	0.206	0.277	0.370	0.443
TH_12	0.133	**0.573**	0.166	0.532
TH_13	−0.054	0.251	0.290	0.181
TH_14	−0.040	0.337	**0.582**	0.574

Factor 1= Engagement in Tactile Treatments

Factor 2 = Communication Facilitation via Touch

Factor 3= Comfort with Touch in Medical settings

h = communalities.

Note: Pattern Matrix Element loadings are rounded to three decimal places.

#### Internal consistency

Cronbach’s alpha was assessed for the for the three factors of the THS indicated by EFA (the 2 low-level loading items were excluded from the analysis). The internal consistency for Engagement in Tactile Treatments and the Communication Facilitation via Touch was high (*α* = 0.816 and *α* = 0.804 respectively). Comfort with Touch in Medical settings showed a lower consistency (*α* = 0.636) compared to the other subscales. The three factors will be described as the three THS subscales thereinafter. Please see the “Internal Consistency” section of the Supplemental Materials for the inter-item correlations.

#### Validity

Convergent validity of the three THS subscales was assessed through their relationship with STQ and TEAQ subscales. All subscales of the STQ were used in these analyses. From the TEAQ, Friends and Family Touch, Attitude to Unfamiliar Touch, and Attitude to Self-Care were used. Childhood Touch, Attitude to Intimate Touch and Current Intimate Touch subscales of TEAQ were judged to be less relevant to attitudes to touch in treatment settings and so were not used for validity analysis (see Supplemental Materials Table 3 for the association of these TEAQ subscales with the three THS subscales). Note that lower STQ scores reflect more positive behavior towards touch, while the opposite is true for TEAQ subscales.

**Table 3.** Pearson correlation coefficients (r) between Touch & Health Scale (THS), social touch questionnaire (STQ) and touch experiences and attitudes questionnaire (TEAQ) subscales.

THS subscales	STQ subscales				TEAQ subscales	
	Liking of familiar Physical touch	Liking of Public Physical touch	Dislike of Physical touch	Attitude to self-care	Attitude to unfamiliar touch	Friends and family touch
Engagement in tactile treatments	−0.592*	−0.432*	−0.375*	0.271*	0.335*	0.334*
Communication facilitation via touch	−0.391*	−0.449*	−0.358*	0.080*	0.364*	0.262*
Comfort with touch in medical settings	−0.322*	−0.354*	−0.422*	−0.016	0.375*	0.203*

Negative correlations with STQ subscales indicate good convergent validity of the THS.

Positive correlations with TEAQ subscales indicate good convergent validity of the THS.

*p < 0.001.

The THS and STQ subscales showed significant negative associations. The strongest correlation between Engagement in Tactile Treatments and STQ subscales was observed with Liking of Familiar Physical Touch, followed by Liking of Public Physical Touch and lastly by Dislike of Physical Touch. Communication Facilitation via Touch showed the strongest association with Liking of Public Physical Touch, followed by Liking of Familiar Physical Touch and Dislike of Physical Touch. Comfort with Touch in Medical settings associated more strongly with Dislike of Physical Touch, followed by Liking of Public Physical Touch and lastly by Liking of Familiar Physical Touch (see Table 3 for correlation coefficients).

The THS and TEAQ subscales showed significant positive associations. The strongest correlation between Engagement in Tactile Treatments and TEAQ subscales was observed with Attitude to Unfamiliar Touch, and Friends and Family Touch, followed by Attitude to Self-Care. Communication Facilitation via Touch associated primarily with Attitude to Unfamiliar Touch, followed by Friends and Family Touch and by a weaker association with Attitude to Self-Care. Comfort with Touch in Medical settings correlated more strongly with Attitude to Unfamiliar Touch, followed by Friends and Family Touch. Finally, the correlation between Comfort with Touch in Medical settings and Attitude to Self-Care was not significant (*p* = 0.069; see Table 3 for correlation coefficients).

### How do attitudes to touch in treatment settings change as a function of inter-individual differences?

In order to investigate the relationship between inter-individual differences and the three THS subscales indicated by the EFA, Mann-Whitney non-parametric t-tests were used for dichotomous variables (gender and age-group comparisons), because of significant deviations of the THS subscales scores from the normality of distribution. For age group comparisons, nine age groups were created according to 10-years bins (18–19, 20–29, 30–39, 40–49, 50–59, 60–69, 70–79, 80–89, 90–92 years), with paired comparisons conducted between the consecutive age groups. Only groups with sufficient sample sizes (*N* ≥ 10 participants) were included, meaning that the age group 90–92 years was excluded from the analysis due to the small sample size (*N* = 3 participants). Additionally, permutation t-tests were conducted for the comparisons with large sample size differences (sample size 1 ≥ 2 × sample size 2).

Bootstrapped Pearson’s correlations (1000 samples) were performed for the continuous variables. For significant correlations (*p* ≤ 0.003), hierarchical regression analyses were conducted to test the relative contribution of a given individual difference measure (predictor variable) to the dependent variables (THS subscales) while controlling for: age, gender and survey completion date. The control variables were included in Step 1 of the hierarchical regression, and the predictor variable(s) of interest were entered in Step 2. The contribution of the inter-individual differences to the three THS subscales was examined in separate regression analyses.

#### Correlations & man-Whitney tests

##### Age

Bootstrapped Pearson’s correlations showed a significant positive association between the THS subscales and age. In particular, the strongest association was observed with Comfort with Touch in Medical settings (*r* = 0.129, *p* < 0.001), followed by Communication Facilitation via Touch (*r* = 0.117, *p* < 0.001). A weak but statistically significant relationship was observed between ageing and Engagement in Tactile Treatments (*r* = 0.036, *p* < 0.001). Across all correlations, older participants reported more positive attitudes and behaviour towards touch in treatment settings than younger participants.

Mann-Whitney non-parametric tests showed significant differences between age groups in participants’ attitudes and behaviour towards touch in treatment settings. Specifically, attitudes towards Engagement in Tactile Treatments significantly increased with age when comparing 18–19 vs 20–29 age groups (*U* = 18986, *p* < 0.001, *r* = −0.21) and 20–29 vs 30–39 (*U* = 229760.5, *p* < 0.001, *r* = −0.11). Communication Facilitation via Touch increased in later stages of adulthood; significant differences were found between 50–59 vs 60–69 (*U* = 5899969, *p* < 0.001, *r* = −0.06) and 60–69 vs 70–79 (*U* = 3411866, *p* < 0.001, *r* = −0.05). Comfort with Touch in Medical settings showed an almost constant increase across age groups. There were significant differences between 18–19 vs 20–29 (*U* = 20099, *p* < 0.001,*r* = −0.19), 20–29 vs 30–39 (*U* = 237500, *p* < 0.001, *r* = −0.09), 40–49 vs 50–59 (*U* = 2272620.5, *p* < 0.001, *r* = −0.04) and 50–59 vs 60–69 (*U* = 5955613, *p* < 0.001, *r* = −0.06; see Supplemental Materials Table 4 for mean scores and Supplemental Materials Table 5 for additional details of the age group comparisons). All the non-significant and significant observations were confirmed by permutation t-tests.

##### Gender

Mann-Whitney non-parametric tests showed significant differences between males and females when comparing their scores for each of the three THS subscales. Women showed increased positive attitudes towards Engagement in Tactile Treatments compared to men (*U* = 12599289.5, *p* < 0.001, *r* = −0.063). However, women did not perceive that they communicate better while being touched in treatment settings to the degree men did (*U* = 11227136.5, *p* < 0.001, *r* = −0.137). Finally, women showed decreased Comfort with Touch in Medical settings compared to men (*U* = 11365657.5, *p* < 0.001, *r* = −0.130; see Supplemental Materials Table 6 for mean scores). Permutation t-test validated the significance of these observations.

##### Psychological traits

The association of the THS subscales with the psychological traits of interest are shown in Table 4. Engagement in Tactile Treatments correlated positively with agreeableness, conscientiousness, extraversion, openness, body acceptance and interoceptive accuracy and negatively with neuroticism and avoidant attachment. However, Engagement in Tactile Treatments did not correlate with anxious attachment (*p* = 0.45). Communication Facilitation via Touch correlated positively with agreeableness, extraversion, openness, anxious attachment, body acceptance and interoceptive accuracy and negatively with neuroticism and avoidant attachment, but it did not correlate with conscientiousness (*p* = 0.81). Finally, Comfort with Touch in Medical settings correlated positively with agreeableness, conscientiousness, extraversion, openness, body acceptance and interoceptive accuracy and negatively with neuroticism, avoidant and anxious attachment. The correlation analyses that were run between the 16-items interoceptive accuracy scores (5 items relevant to COVID-19 symptomatology were excluded) and the THS subscale scores showed positive associations, similar to the association of the 21-items interoceptive accuracy scale scores and the THS subscale scores (see Supplemental Materials Table 7).

**Table 4.** Pearson’s Correlation coefficients (r) between the touch & health scale (THS) subscales and the traits of personality (BFI-S), attachment style (ECR-12), body acceptance (DBIQ) and interoceptive accuracy (IAS).

Instrument		THS subscales		
		Engagement in tactile treatments	Communication facilitation via touch	Comfort with touch in medical settings
BFI-S	Agreeableness	0.125*	0.101*	0.110*
	Conscientiousness	0.071*	0.002	0.050*
	Extraversion	0.289*	0.182*	0.189*
	Neuroticism	−0.125*	−0.072*	−0.155*
	Openness	0.134*	0.122*	0.089*
ECR-12	Avoidant attachment	−0.199*	−0.139*	−0.189*
	Anxious attachment	0.006	0.095*	−0.067*
DBIQ	Body acceptance	0.149*	0.118*	0.204*
IAS	Interoceptive accuracy	0.114*	0.055*	0.070*

*p < 0.001.

### Regression analyses

#### Personality traits

##### Engagement in tactile treatments

The overall regression model predicted participants’ Engagement in Tactile Treatments scores (*F*(8, 12275) = 157.76, *p* < 0.001) by explaining 9.3% of the variance (see Table 5 for the description of coefficients). There was a significant R square change in Model 1 (*ΔR*^2^ = 0.004, *ΔF*(3,12280) = 15.44, *p* < 0.001) and a significant R square change in Model 2 (*ΔR*^2^ = 0.089, *ΔF*(5,12275) = 242.25, *p* < 0.001). Among the five personality traits, extraversion was the strongest positive predictor of Engagement in Tactile Treatments scores (*b* = 0.250, *p* < 0.001), indicating that the higher one is in extraversion, the more likely one is to engage in tactile treatments. Less strong, but significant positive predictors were agreeableness (*b* = 0.073, *p* < 0.001) and openness (*b* = 0.045, *p* < 0.001). Neuroticism was a weak negative predictor of Engagement in Tactile Treatments scores (*b* = −0.038, *p* < 0.001), while conscientiousness was not a significant predictor of the model (*b* = −0.003, *p* = 0.776).

**Table 5.** Summary of hierarchical regression analysis for personality traits predicting Engagement in Tactile Treatments scores, r indicates zero order correlations.

Predictor variables	B	SE(B)	Beta	t	Sig.	R
**Model 1**						
*Age*	0.011	0.002	0.040	4.405	<0.001	
*Gender*	0.425	0.078	0.049	5.445	<0.001	
*Completion Date*	0.001	0.002	0.003	0.284	0.776	
**Model 2**						
*Age*	0.003	0.002	0.011	1.312	0.190	
*Gender*	0.145	0.077	0.017	1.888	0.059	
*Completion Date*	0.001	0.002	0.003	0.389	0.697	
*Extraversion*	0.233	0.009	0.250	25.965	<0.001	0.289*
*Agreeableness*	0.100	0.012	0.073	8.206	<0.001	0.125*
*Openness*	0.056	0.011	0.045	4.917	<0.001	0.134*
*Neuroticism*	−0.034	0.008	−0.038	−4.008	<0.001	−0.125*
*Conscientiousness*	−0.003	0.012	−0.003	−0.284	0.776	0.071*

*p < 0.001.

##### Communication facilitation via touch

The overall regression model predicted participants’ Communication Facilitation via Touch scores (*F*(7, 12278) = 147.13, *p* < 0.001) by explaining 7.7% of the variance (see Supplemental Materials Table 8 for the description of coefficients). There was a significant R square change in Model 1 (*ΔR*^2^ = 0.030, *ΔF*(3,12282) = 127.35, *p* < 0.001) and a greater R square change in Model 2 (*ΔR*^2^ = 0.047, *ΔF*(4,12278) = 157.102, *p* < 0.001). Among the four personality traits extraversion was the strongest predictor, showing a significant positive association with Communication Facilitation via Touch scores (*b* = 0.174, *p* < 0.001). Less strong, but significantly positive predictors were agreeableness (*b* = 0.080, *p* < 0.001) and openness (*b* = 0.056, *p* < 0.001). Neuroticism was a weak predictor of Communication Facilitation via Touch scores (*b* = −0.031, *p* < 0.001).

##### Comfort with touch in medical settings

The overall regression model predicted participants’ Comfort with Touch in Medical settings scores (*F*(8, 12275) = 133.65, *p* < 0.001) by explaining 8% of the variance (see Table 6 for the description of coefficients). There was a significant R square change in Model 1 (Δ*R*^2^ = 0.03, *ΔF*(3, 12280) = 127.94, *p* < 0.001) and a greater R square change in Model 2 (*ΔR*^2^ = 0.05, *ΔF*(5,12275) = 132.96, *p* < 0.001). Among the five personality traits, extraversion was the strongest predictor, showing a significant positive association with Comfort with Touch in Medical settings scores (*b* = 0.160, *p* < 0.001). Less strong, but significant predictors were agreeableness (*b* = 0.084, *p* < 0.001) and Neuroticism (*b* = −0.062, *p* < 0.001). Openness (*b* = 0.019, *p* = 0.035) and conscientiousness (*b* = −0.003, *p* = 0.704) were not significant predictors of the model.

**Table 6.** Summary of the hierarchical regression analysis for personality traits predicting comfort with touch in medical settings scores, r indicates zero order correlations.

Predictor variables	B	SE(B)	Beta	t	Sig.	r
**Model 1**						
*Age*	0.020	0.001	0.123	13.762	<0.001	
*Gender*	−0.591	0.045	−0.116	−13.051	<0.001	
*Completion Date*	−0.001	0.001	−0.008	−0.907	0.364	
**Model 2**						
*Age*	0.016	0.001	0.098	11.114	<0.001	
*Gender*	−0.688	0.045	−0.135	−15.163	<0.001	
*Completion Date*	−0.001	0.001	−0.007	−0.841	0.400	
*Extraversion*	0.088	0.005	0.160	16.522	<0.001	0.188*
*Agreeableness*	0.068	0.007	0.084	9.366	<0.001	0.110*
*Openness*	0.014	0.007	0.019	2.114	0.035	0.089*
*Neuroticism*	−0.033	0.005	−0.062	−6.568	<0.001	−0.155*
*Conscientiousness*	−0.003	0.007	−0.003	−0.381	0.704	0.050*

*p < 0.001.

#### Attachment style

##### Engagement in tactile treatments

The overall regression model predicted participants’ Engagement in Tactile Treatments scores (*F*(4, 12075) = 139.91, *p* < 0.001) by explaining 4.4% of the observed variance in the Engagement in Tactile Treatments scores (see Table 7 for the description of coefficients). There was a significant R square change in Model 1 (*ΔR*^2^ = 0.004, *ΔF*(3,12076) = 15.45, *p* < 0.001) and a greater R square change in Model 2 (*ΔR*^2^ = 0.04, *ΔF*(1,12075) = 511.33, *p* < 0.001). This was linked to a significant negative association between Engagement in Tactile Treatments scores and avoidant attachment (*b* = −0.202, *p* < 0.001), indicating that the more avoidant behaviour one has, the more likely one is to avoid tactile treatments.

**Table 7.** Summary of the hierarchical regression analysis for avoidant attachment style predicting Engagement in Tactile Treatments scores, r indicates zero order correlations.

Predictor variables	B	SE(B)	Beta	t	Sig.	r
**Model 1**						
*Age*	0.011	0.002	0.041	4.526	<0.001	
*Gender*	0.421	0.079	0.049	5.360	<0.001	
*Completion Date*	0.000	0.002	0.002	0.231	0.817	
**Model 2**						
*Age*	0.014	0.002	0.053	5.931	<0.001	
*Gender*	0.407	0.077	0.047	5.287	<0.001	
*Completion Date*	0.001	0.002	0.005	0.602	0.547	
*Avoidant Attachment*	−0.101	0.004	−0.202	−22.613	<0.001	−0.199*

*p < 0.001.

##### Communication facilitation via touch

The overall regression model predicted participants’ Communication Facilitation via Touch scores (*F*(5, 12073) = 178.81, *p* < 0.001) by explaining 6.9% of the observed variance in the Communication Facilitation via Touch scores (see Supplemental Materials Table 9 for the description of coefficients). There was a significant R square change in Model 1 (*ΔR*^2^ = 0.030, *ΔF*(3,12075) = 126.502, *p* < 0.001) and a greater R square change in Model 2 (*ΔR*^2^ = 0.038, *ΔF*(2,12073) = 249.46, *p* < 0.001). This was linked to a significant negative association between participants’ Communication Facilitation via Touch scores and avoidant attachment (*b* = −0.162, *p* < 0.001), indicating that the more avoidant behaviour one has, the more likely it is to feel uncomfortable with talking while being touched in treatment settings. Finally, there was a positive correlation with the anxious attachment (*b* = 0.132, *p* < 0.001).

##### Comfort with touch in medical settings

The overall regression model predicted participants’ Comfort with Touch in Medical settings scores (*F*(5, 12073) = 183.13, *p* < 0.001) by explaining 7% of the observed variance in the Comfort with Touch in Medical settings scores (see Table 8 for the description of coefficients). There was a significant R square change in Model 1 (*ΔR*^2^ = 0.031, *ΔF*(3,12075) = 126.85, *p* < 0.001) and a greater R square change in Model 2 (*ΔR*^2^ = 0.040, *ΔF*(2,12073) = 259.41, *p* < 0.001). This was linked to a significant negative association between Comfort with Touch in Medical settings scores and avoidant attachment (*b* = −0.195, *p* < 0.001), indicating that the more avoidant behaviour one has, the more likely one is to avoid touch in medical settings. Finally, anxious attachment was a weak, but significant predictor of Comfort with Touch in Medical settings scores (*b* = −0.028, *p* = 0.002).

**Table 8.** Summary of the hierarchical regression analysis for avoidant and anxious attachment styles predicting Comfort with Touch in Medical settings scores, r indicates zero order correlations.

Predictor variables	B	SE(B)	Beta	t	Sig.	r
**Model 1**						
*Age*	0.019	0.001	0.122	13.594	<0.001	
*Gender*	−0.596	0.046	−0.177	−13.084	<0.001	
*Completion Date*	−0.001	0.001	−0.007	−0.767	0.443	
**Model 2**						
*Age*	0.021	0.001	0.130	14.608	<0.001	
*Gender*	−0.603	0.045	−0.119	−13.519	<0.001	
*Completion Date*	0.000	0.001	−0.004	−0.427	0.669	
*Avoidant Attachment*	−0.058	0.003	−0.195	−22.065	<0.001	−0.189*
*Anxious Attachment*	−0.007	0.002	−0.028	−3.092	0.002	−0.067*

*p < 0.001.

#### Body acceptance

##### Engagement in tactile treatments

The overall regression model predicted participants’ Engagement in Tactile Treatments scores (*F*(4, 12048) = 82.72, *p* < 0.001) by explaining 2.6% of the observed variance in the Engagement in Tactile Treatments scores (see Supplemental Materials Table 10 for the description of coefficients). There was a significant R square change in Model 1 (*ΔR*^2^ = 0.004, *ΔF*(3,12049) = 14.44, *p* < 0.001) and a greater R square change in Model 2 (*ΔR*^2 ^= 0.023, *ΔF*(1,12048) = 286.51, *p* < 0.001). This was linked to a significant positive association between Engagement in Tactile Treatments scores and body acceptance (*b* = 0.155, *p* < 0.001), indicating that the more comfortable one feels with their body, the more likely one is to engage in tactile treatments.

##### Communication facilitation via touch

The overall regression model predicted participants Communication Facilitation via Touch scores (*F*(4, 12048) = 121.41, *p* < 0.001) by explaining 3.8% of the observed variance in the Communication Facilitation via Touch scores (see Supplemental Materials Table 11 for the description of coefficients). There was a significant R square change in Model 1 (*ΔR*^2^ = 0.031, *ΔF*(3,12049) = 127.65, *p* < 0.001) and a significant R square change in Model 2 (*ΔR*^2^ = 0.008, *ΔF*(1,12048) = 99.52, *p* < 0.001). This was linked to a significant and positive association between Communication Facilitation via Touch scores and body acceptance (*b* = 0.090, *p* < 0.001), indicating that the more comfortable one feels with their body, the more likely one is to communicate their thoughts while being touched in treatment settings.

##### Comfort with touch in medical settings

The overall regression model predicted participants’ Comfort with Touch in Medical settings scores (F(4, 12048) = 199.39, *p* < 0.001) by explaining 6.2% of the observed variance in the Comfort with Touch in Medical settings scores (see Supplemental Materials Table 12 for the description of coefficients). There was a significant R square change in Model 1 (*ΔR*^2^ = 0.031, *ΔF*(3,12049) = 128.40, *p* < 0.001), and also in Model 2 (*ΔR*^2^ = 0.031, *ΔF*(1,12048) = 399.61, *p* < 0.001). This was linked to a significant positive association between Comfort with Touch in Medical settings scores and body acceptance (*b* = 0.204, *p* < 0.001), indicating that the more comfortable one feels with their body, the more likely one is to feel comfortable being touched in medical settings.

#### Interoceptive accuracy

##### Engagement in tactile treatments

The overall regression model predicted participants’ Engagement in Tactile Treatments scores (*F*(4, 12029) = 46.79, *p* < 0.001) by explaining 1.5% of the observed variance in the Engagement in Tactile Treatments scores (see Supplemental Materials Table 13 for the description of coefficients). There was a significant R square change in Model 1 (*ΔR*^2^ = 0.004, *ΔF*(3,12030) = 14.49, *p* < 0.001) and a greater R square change in Model 2 (*ΔR*^2^ = 0.012, *ΔF*(1,12029) = 143.181, *p* < 0.001). This was linked to a significant, positive association between participants’ Engagement in Tactile Treatments scores and the interoceptive accuracy scores (*b* = 0.109, *p* < 0.001), indicating the more precisely one perceives the signals of their internal body the more likely one is to engage in tactile treatments.

##### Communication facilitation via touch

The overall regression model predicted participants’ Communication Facilitation via Touch scores (*F*(4, 12029) = 105.46, *p* < 0.001) by explaining 3.4% of the observed variance in the Communication Facilitation via Touch scores (see Supplemental Materials Table 14 for the description of coefficients). There was a significant R square change in Model 1 (*ΔR*^2^ = 0.031, *ΔF*(3,12030) = 127.298, *p* < 0.001) and a smaller R square change in Model 2 (*ΔR*^2^ = 0.003, *ΔF*(1,12029) = 38.758, *p* < 0.001). This was linked to a significant positive association between Communication Facilitation via Touch scores and the interoceptive accuracy scores (*b* = 0.056, *p* < 0.001), indicating that the more precisely one perceives the signals of their internal body, the more likely one is to feel comfortable with talking while being touched in treatment settings.

##### Comfort with touch in medical settings

The overall regression model predicted participants’ Comfort with Touch in Medical settings scores (*F*(4, 12029) = 111.72, *p* < 0.001) by explaining 3.5% of the observed variance in the Comfort with Touch in Medical settings scores (see Supplemental Materials Table 15 for the description of coefficients). There was a significant R square change in Model 1 (*ΔR*^2^ = 0.031, *ΔF*(3,12030) = 128.29, *p* < 0.001) and a smaller R square change in Model 2 (*ΔR*^2^ = 0.005, *ΔF*(1,12029) = 69.10, *p* < 0.001). This was linked to a significant positive association between Comfort with Touch in Medical settings scores and interoceptive accuracy scores (*b* = 0.07, *p* < 0.001), indicating that the more precisely one perceives the signals of their internal body the more likely one is to feel comfortable being touched in medical settings.

### How do attitudes and experiences of touch in treatment settings relate to mental wellbeing and loneliness?

To investigate the relationship between touch attitudes in treatment settings and wellbeing, bootstrapped Pearson’s correlations (1000 samples) were performed between the THS subscales scores and the two measurements of wellbeing (mental wellbeing measured by SWEMWBS and loneliness measured by the UCLA Loneliness scale). To identify the predictive value of touch attitudes in treatment settings on mental wellbeing and loneliness, hierarchical regression analyses were also performed for each THS subscale separately, following the same approach as previously described in the previous Results section (“How do attitudes to touch in treatment settings change as a function of inter-individual differences?”).

#### Correlations

Bootstrapped Pearson’s correlations showed significant positive associations between the THS subscales and wellbeing (loneliness and mental wellbeing). Loneliness showed the strongest association with Engagement in Tactile Treatments (r = −0.214, *p* < 0.001), followed by Comfort with Touch in Medical settings (*r* = −0.170, *p* < 0.001). A weak, but significant relationship, was observed between loneliness and Communication Facilitation via Touch (*r* = −0.079, *p* < 0.001). Similarly, mental wellbeing showed the strongest association with Engagement in Tactile Treatments (*r* = 0.138, *p* < 0.001), followed by Comfort with Touch in Medical settings (*r* = 0.137, *p* < 0.001). A weak, but significant relationship, was observed between mental wellbeing and Communication Facilitation via Touch (*r* = 0.042, *p *< 0.001). Please see Supplemental Materials Table 7 for additional analyses relevant to loneliness scores measured by the 4-items and 8-items UCLA.

### Regressions

#### Mental wellbeing (SWEMWBS)

The overall regression model predicted participants’ mental wellbeing scores (*F*(6, 12282) = 98.16, *p* < 0.001) by explaining 4.5% of the variance in the mental wellbeing scores (see Supplemental Materials Table 16 for the description of coefficients). There was a significant R square change in Model 1 (*ΔR*^2^ = 0.019, *ΔF*(3,12285) = 78.12, *p* < 0.001), but a greater R square change in Model 2 (*ΔR*^2^ = 0.027, *ΔF*(3,12282) = 116.01, *p* < 0.001). The strongest predictor was Engagement in Tactile Treatments (*b* = 0.123, *p* < 0.001), indicating that Engagement in Tactile Treatments predicts greater wellbeing. Less strong, but a significant and positive predictor was Comfort with Touch in Medical settings (*b* = 0.105, *p* < 0.001). Finally, Communication Facilitation via Touch was a weak negative predictor of mental wellbeing (*b* = −0.70, *p* < 0.001). Thus, individuals who indicated they are more talkative during tactile treatments are more likely to report lower mental wellbeing.

#### Loneliness (UCLA-loneliness)

The overall regression model predicted participants’ loneliness scores (*F*(6, 12034) = 159.53, *p* < 0.001) by explaining 7.3% of the variance in loneliness scores (see Table 9 for the description of coefficients). There was a significant R square change in Model 1 (*ΔR*^2^ = 0.018, *ΔF*(3,12037) = 72.86, *p* < 0.001), but a greater R square change in Model 2 (*ΔR*^2^ = 0.056, *ΔF*(3,12034) = 241.821, *p* < 0.001). The strongest predictor was Engagement in Tactile Treatments (*b* = −0.177, *p* < 0.001), followed by Comfort with Touch in Medical settings (*b* = −0.129, *p* < 0.001), indicating that engaging in tactile treatments and feeling comfortable with touch in medical settings lead to reduced loneliness. Finally, Communication Facilitation via Touch was a weak predictor of loneliness (*b* = 0.048, *p* < 0.001), indicating that participants who indicated that they feel comfortable at communicating in treatment settings are more likely to report experiencing feelings of loneliness.

**Table 9.** Summary of the hierarchical regression analysis for touch & health scale subscales predicting loneliness scores, r indicates zero order correlations.

Predictor variables	B	SE(B)	Beta	t	Sig.	r
**Model 1**						
*Age*	−0.024	0.007	−0.030	−3.307	0.001	
*Gender*	−3.289	0.227	−0.131	−14.456	<0.001	
*Completion Date*	0.010	0.005	0.016	1.817	0.069	
**Model 2**						
*Age*	−0.010	0.007	−0.012	−1.397	0.163	
*Gender*	−3.321	0.226	−0.132	−14.684	<0.001	
*Completion Date*	0.010	0.005	0.017	1.945	0.052	
*ETT*	−0.514	0.031	−0.177	−16.514	<0.001	−0.214*
*CFT*	0.145	0.032	0.048	4.479	<0.001	−0.079*
*CTM*	−0.640	0.050	−0.129	−12.750	<0.001	−0.170*

*p< 0.001.

ETT= Engagement in Tactile Treatments.

CFT= Communication Facilitation via Touch.

CTM= Comfort with Touch in Medical settings.

## Discussion

The current study sought to investigate inter-individual differences that contribute to attitudes towards touch in treatment settings and how attitudes towards touch in treatment settings are associated with wellbeing. To do so, we developed and tested a novel measure of touch in treatment settings, entitled ‘The Touch & Health Scale’ (THS). The original THS was constructed by 14 items, and following the exploratory factor analysis, it was reduced to 12-items. The 12-item instrument was found to be a reliable and valid measure of three aspects of participants’ attitudes and behaviour, namely Engagement in Tactile Treatments, Communication Facilitation via Touch in treatment settings and Comfort with Touch in Medical settings. The validation with the Social Touch and the Touch Experiences and Attitudes scales showed that touch attitudes in treatment settings relate to the patterns of individuals’ general day-to-day attitudes towards touch. Significant changes in THS subscale scores were observed according to inter-individual differences in demographic characteristics and psychological traits. Among the studied inter-individual differences, increased extraversion and low avoidant attachment were the strongest predictors of participants’ positive approach to touch in treatment settings. Finally, positive attitudes to touch in treatment settings were associated with high mental wellbeing and reduced loneliness. Overall, the study supports the need for a person-centred approach when considering touch in treatment settings, as some individuals might be less comfortable with touch than others. In the following sections, we describe in more detail the outcomes of the present study, and we comment on their relation to previous literature and the approaches to future research.

## The psychometrics of the THS

The THS is an instrument to measure attitudes to touch in treatment settings via the three subscales, as identified by the factor analysis. The Engagement in Tactile Treatments subscale describes individuals seeking tactile treatments such as massage. Comfort with Touch in Medical settings subscale identifies individuals who feel comfortable being touched and seen by their doctor in a treatment session. Both Engagement in Tactile Treatments and Comfort with Touch in Medical settings subscales could be used as screening tools to guide therapists on approaching their patients better. Communication Facilitation via Touch subscale identifies individuals that feel more relaxed while being touched in treatment settings, aiding their oral communication with their therapist/carer. Facilitation of verbal communication is an essential element in treatment settings, and identification of means that can encourage this behaviour, such as haptic sensations, are valuable. In nursing, patients report that non-judgemental listening by the nurse and nurse’s talking prior to or during applying touch aids building a trusting relationship. In addition, nurses acknowledge that patient’s talkative behaviour facilitates their understanding of the patient’s needs (Fleischer et al., 2009; O’Lynn and Krautscheid, 2011).

The three THS subscales showed acceptable to good reliability in terms of internal consistency (according to Cronbach’s α = 0.60 cut-off), and according to criteria reported in the literature (Ponterotto and Ruckdeschel, 2007; Taber, 2018; Van Griethuijsen et al., 2015; Vaske et al., 2017). The THS subscales correlated significantly with the Social Touch Questionnaire (STQ) and Touch Experiences and Attitudes Questionnaire (TEAQ) subscales, demonstrating convergent validity with other measures of touch attitudes.

The only observation that did not show good convergent validity was the association between Comfort with touch in Medical settings and the TEAQ subscale of Attitude to Self-Care. As Attitude to Self-Care is the subscale directly relevant to measuring attitudes to touch in the context of health, a convergent relationship was predicted. In the original paper of Trotter et al. (2018), the factors Attitude to Self-Care and Attitude to Unfamiliar Touch showed the weakest correlation compared to the rest of the factor correlations. Therefore, it is possible that the Self-Care construct which measures self-touch and individual tactile experiences, does not align with touch attitudes in settings where unfamiliar touch is involved (e.g., touch by the therapist in medical settings). The latter observation could explain the non-significant association between Attitude to Self-Care and Comfort with touch in Medical settings in our study. Further, Attitude to Self-Care refers to tactile experiences intended to be therapeutic or rewarding, such as baths and beauty treatments. In contrast, Comfort with touch in Medical settings refers to touch that is not only therapeutic, but can be used for diagnostic purposes or as a non-verbal cue, leading to possible discomfort.

## Individual differences in THS

The association of inter-individual differences with touch attitudes in treatment settings followed several of our predictions. Firstly, in line with our hypothesis, women were more positive about engaging in tactile treatments than men. This observation aligns with prior research that reports more positive responses to touch in women than men (Trotter et al., 2018; Webb and Peck, 2015) and precisely their positive attitude to self-care (Trotter et al., 2018), as tactile treatments can be considered a self-care practice. However, women’s positive attitude to touch in treatment settings seems specific to tactile treatments, as men showed more comfort with touch in medical settings and were more open to communication while being touched than the women in our study. This finding is not surprising, considering that receiving touch from a medical professional may mirror power differences and gender inequalities ingrained in society, that often place women in a vulnerable position and at risk of sexual assault (Alyn, 1988; Twigg et al., 2011; Wearn et al., 2020). Literature in medical settings recognises that touch is a mean of exerting power and elaborates on ways to address this issue (Kelly et al., 2018). However, touch is not always necessary in medical settings, and the patient might be unfamiliar with the medical professional delivering the touch. Our finding supports that women might be more uncomfortable receiving unexpected and unfamiliar touch, particularly from men, compared to their male counterparts (Guerrero and Andersen, 1994; Trotter et al., 2018). Future research may wish to examine whether an increased probability of feeling threatened by unfamiliar touch may explain decreased comfort with receiving physical touch in medical settings, and less open communication while receiving touch.

Age differences were also linked with altered attitudes to touch in treatment settings, with changes in participants’ behaviour occurring in specific age ranges. Engagement in Tactile Treatments increased during early adulthood (18–39 years) and was stabilised in the early middle age (after 40 years). Comfort with Touch in Medical settings progressively increased from early adulthood to the later adult stages with only one age period, between 30–49 years, remaining stable. These findings are in line with Webb and Peck (2015), which showed comfort with interpersonal touch increased by age in a sample ranging from 18 to 76 years. Communication Facilitation in Treatment settings did not change significantly until reaching 50 years, and it continued changing towards more positive scores until 79 years. Here, it is worth mentioning that decline in health and more frequent use of healthcare facilities during ageing is reported in the literature (Alemayehu and Warner, 2004; Chechulin et al., 2014; Kelly et al., 2016). This evidence indicates the increased need to interact with medical professionals in adults aged over 50, which could explain the increase of communicative behaviour in treatment settings after the age of 50 and in later life stages in our study. Despite the mixture of previous findings about the effect of age on touch attitudes, our findings support that attitudes to touch in treatment settings progress towards more positive scores during ageing.

Several psychological traits of interest were significantly associated with the THS subscales, but only a few were strong predictors for touch attitudes in treatment settings. More specifically, positive attitudes across all THS subscales were associated with the following traits: high extraversion, high openness, high agreeableness, high body acceptance, high interoceptive ability, low neuroticism, and low avoidant attachment. Among those traits, high extraversion and low avoidant attachment style showed the strongest predicting value. Our findings are in line with prior evidence showing that extraverted individuals are comfortable with touch when interacting with other people (Fuller et al., 2011), and they have positive attitudes towards massage treatments (Moyer and Rounds, 2009). In contrast, individuals with high traits of avoidant attachment were associated with less positive attitudes to touch in treatment settings, suggesting that the observed avoidance of interpersonal proximity (Kaitz et al., 2004) is reflected in treatment settings.

It is also worth noting that high body acceptance scores showed a moderately good predictive value for comfort with touch in treatment settings. To our knowledge, the link between satisfaction with one’s body image and touch from a stranger in a typical sample is understudied and focused on the therapeutic aspect of touch (e.g. massage treatment, Dunigan et al., 2011; Espí-López et al., 2020). Further investigation on how body image affects the different forms of touch attitudes (e.g. the distinction between therapeutic and diagnostic) in a range of settings is essential.

Conscientiousness and anxious attachment were the psychological traits that showed different association patterns with THS subscales compared to our hypotheses. Conscientious individuals showed more positive attitudes to touch in tactile treatments, and they were more comfortable with receiving touch in treatment settings. Still, their communication in treatment settings was not affected by touch any more than for less conscientious individuals. Anxious attachment was not associated with the desire to get involved in tactile treatments. However, an anxious attachment style was associated with reduced Comfort with Touch in Medical settings. Prior literature supports that pleasant touch (CT-afferent) from a stranger or romantic partner facilitates pain reduction in individuals with increased anxious attachment style (Krahé et al., 2016; Von Mohr et al., 2018) and that tactile treatments are a means of pain relief (Thrane and Cohen, 2014). In agreement with the previous observation, we hypothesised that anxious attachment trait would be linked with more positive attitudes to touch in treatment settings because of the beneficial effect of touch on health. However, Krahé et al. (2018) found that individuals with higher anxious attachment were less sensitive to discrimination of optimal-CT touch (pleasant touch) versus non-Optimal CT-Touch (control condition) that was delivered by a stranger (experimenter). This may suggest that familiarity with the person giving touch is an essential factor that could influence touch experiences in treatment settings for people high in anxious attachment. Another possible explanation for our results could be that the reported positive effect of touch in anxiously attached individuals is specific to pain, and its generalisability to other forms and contexts of touch (e.g. in treatment settings) requires further investigation.

## THS and wellbeing

Higher wellbeing was associated with increased Engagement in Tactile Treatments and Comfort with Touch in Medical settings scores, while a weaker association was observed with Communication Facilitation via Touch. The high scores of Engagement in Tactile Treatments and Comfort with Touch in Medical settings were the strongest predictors of greater mental wellbeing and reduced loneliness. These findings suggest that not only tactile treatments, but in general, receiving touch in treatment settings, has a positive link with an individual’s wellbeing. In prior literature, therapeutic touch (e.g. massage) is known for its health benefits on the physical and psychological wellbeing (e.g., Field, 2019). In addition, patients report a sense of grounding and safety when they are touched by their psychoanalyst (Pinson, 2002). Patients also report feelings of comfort and warmth when they are touched by a nurse (Gleeson and Timmins, 2004). Our findings add to this body of literature and support that the different touch forms in treatment settings can benefit a patient’s wellbeing and feelings of loneliness.

## Conclusion

The present study has demonstrated that the THS is a robust instrument to assess attitudes to touch in treatment settings with subscales’ internal consistency ranging between Cronbach’s α = 0.636 to 0.816 and convergent validity showing significant correlations (*p* < 0.001) with the Touch Experiences and Attitudes (TEAQ; Pearson’s r = 0.080 to 0.375) and the Social Touch Questionnaires (STQ; Pearson’s r =.−0.592 to −0.322). The findings also show that attitudes to touch in treatment settings map to general day-to-day attitudes towards touch. In addition, high extraversion and low avoidant attachment traits contribute to patients’ positive touch attitudes in treatment settings. The benefit of adopting positive attitudes to touch in treatment settings was highlighted by their predictive value of greater mental wellbeing and reduced loneliness. However, this beneficial influence may change according to individual differences, suggesting a person-centred approach in treatment settings. The THS may therefore offer a valuable pre-screening tool for medical experts on how to approach their patients better, guiding their decision-making and the design of their therapeutic approaches. For instance, in psychotherapy there are therapeutic practices that involve touch between the therapist and patient, but it is not always clear if and in what degree these tactile practices are welcomed and beneficial for the patient (Giannone, 2015; Kelly et al., 2018). Finally, THS might be used in future research to further investigate individuals’ attitudes to touch in treatment settings, such as dynamic changes in one’s attitudes to touch over time.

## Supplemental Material

Supplemental material - Assessing individual differences in attitudes towards touch in treatment settings: Introducing the touch & health scale Click here for additional data file. Supplemental material for Assessing individual differences in attitudes towards touch in treatment settings: Introducing the touch & health scale by Aikaterini Vafeiadou, Natalie C Bowling, Claudia Hammond and Michael J Banissy in Health Psychology Open

## Note

1 The research was pre-registered with Open Science Framework (OSF). The pre-registration document is available in OSF storage under the name “Touch & Health Preregistration.pdf”. In addition, all the files relevant to the data analysis are included in the OSF storage. Please note that the “README.pdf” is the guide that describes the content of the OSF storage files.
