# Supplementary material for: Assessing individual differences in attitudes towards touch in treatment settings: Introducing the touch & health scale
Source: Health Psychol Open. 2022 Nov 14;9(2):20551029221137008. doi: 10.1177/20551029221137008 (PMC9682164; doi:10.1177/20551029221137008)
Supplement: Supplemental material - Assessing individual differences in attitudes towards touch in treatment settings: Introducing the touch & health scale [file sj-pdf-1-hpo-10.1177_20551029221137008.pdf]

Supplemental Material

Demographic Information

Table 1. The sample size and the demographic characteristics of the participants that were included in the analysis are presented below per each scale/subscale of interest. The samples include UK healthy participants who replied to all the 14 items of the THS and to at least 80% of the scales/subscales. ± indicates Standard Deviation.

Scales/Subscales	Sample Size	Gender Ratio	Mean Age
STQ_DPT	12,273	9,334 F : 2,939 M	56.56 ± 13.60
STQ_LFPT	12,257	9,319 F : 2,938 M	56.55 ± 13.60
STQ_LPPT	12,285	9,342 F : 2,943 M	56.57 ± 13.60
TEAQ_ChT	12,241	9,307 F : 2,934 M	56.60 ± 13.59
TEAQ_FFT	12,246	9,311 F : 2,935 M	56.59 ± 13.59
TEAQ_CIT	12,236	9,303 F : 2,933 M	56.59 ± 13.59
TEAQ_AIT	12,235	9,304 F : 2,931 M	56.59 ± 13.59
TEAQ_ASC	12,239	9,308 F : 2,931 M	56.60 ± 13.59
TEAQ_AUT	12,246	9,311 F : 2,935 M	56.59 ± 13.59
Mental Wellbeing	12,289	9,346 F : 2,943 M	56.57 ± 13.60
Social Wellbeing (Loneliness)	12,041	9,154 F : 2,887 M	56.66 ± 13.54

Avoidant Attachment	12,080	9,177 F : 2,903 M	56.58 ± 13.57
Anxious Attachment	12,086	9,183 F : 2,903 M	56.59 ± 13.57
Agreeableness	12,288	9,344 F : 2,944 M	56.57 ± 13.60
Conscientiousness	12,287	9,343 F : 2,944 M	56.57 ± 13.60
Extraversion	12,290	9,345 F : 2,945 M	56.57 ± 13.60
Openness	12,290	9,345 F : 2,945 M	56.57 ± 13.60
Neuroticism	12,288	9,343 F : 2,945 M	56.57 ± 13.60
Interoceptive Accuracy	12,034	9,144 F : 2,890 M	56.65 ± 13.55
Body Acceptance	12,053	9,167 F : 2,886M	56.64 ± 13.55

DPT = Dislike of Physical Touch, LFPT = Liking of Familiar Physical Touch, LPPT = Liking of Public Physical Touch, ChT= Childhood Touch, FFT = Family and Friends Touch, CIT= Current Intimate Touch, AIT = Attitude to Intimate Touch, ASC = Attitude to Self-Care, AUT = Attitude to Unfamiliar Touch

The psychometric properties of the THS

Table 2. Factor correlation matrix.

Factors	ETT	CFT	CTM
Engagement in Tactile Treatments (ETT)	1.00		
Communication Facilitation via Touch (CFT)	.451	1.00	

Comfort with Touch in Medical settings (CTM)	.376	.380	1.00

Note: Correlation Matrix Elements are rounded to three decimal places.

Internal Consistency

The average intercorrelation coefficient of each THS subscale was calculated (without considering correlations of a given item with itself). The Engagement in Tactile Treatments showed $r_{\text{average inter-item correlation}} = .541$ with variance $\sigma^2 = .015$. The Communication Facilitation via Touch showed $r_{\text{average inter-item correlation}} = .452$ with variance $\sigma^2 = .004$. The Comfort with Touch in Medical settings showed $r_{\text{average inter-item correlation}} = .369$ with variance $\sigma^2 = .001$.

Table 3. Pearson's Correlation coefficients (r) between THS and TEAQ subscales.

THS Subscales	Attitude to Intimate Touch	Current Intimate Touch	Childhood Touch
Engagement in Tactile Treatments	.293*	.136*	.125*
Communication Facilitation via Touch	.262*	.085*	.069*
Comfort with Touch in Medical settings	.247*	.090*	.100*

Positive correlations with TEAQ subscales indicate good convergent validity of the THS.
$* \; p < .001$

Age & Gender differences in the three THS subscales

Table 4. Mean Scores for the THS subscales' scores by age group. ± represents Standard Deviations (SD). High scores indicate more positive attitudes.

Age group (in years)	Number of Participants	Engagement in Tactile Treatments	Communication facilitation via touch	Comfort with touch in Medical Settings

18-19	106	10.74 ± 2.56	12.92 ± 3.21	9.84 ± 2.20
20-29	534	12.50 ± 3.57	13.53 ± 3.58	10.90 ± 2.34
30-39	991	13.30 ± 3.66	13.49 ± 3.56	11.30 ± 2.25
40-49	1514	13.42 ± 3.92	13.74 ± 3.57	11.31 ± 2.22
50-59	3174	13.37 ±3.80	13.94 ± 3.56	11.53 ± 2.18
60-69	4007	13.33 ± 3.61	14.38 ± 3.47	11.78 ± 2.09
70-79	1824	13.36 ± 3.57	14.81 ± 3.44	11.88 ± 2.07
80-89	138	12.82 ± 3.09	14.67 ± 3.53	11.81 ± 1.83

Table 5. U and p values of the Mann-Whitney comparisons between the different age groups.

Comparison of Age Groups	Engagement in Tactile Treatments		Communication Facilitation via Touch		Comfort with touch in Medical settings	
	U	*P-value*	*U*	*P-value*	*U*	*P-value*
18-19 vs 20-29	18986	.00000008*	25146.5	.068513	20099	.000002*
20-29 vs 30-39	229760.5	.00002*	264259	.967011	237500	.000842*
30-39 vs 40-49	729251.5	.235334	723198	.125885	745619	.794180
40-49 vs 50-59	2369487	.441652	2310144	.031955	2272620.5	.002405*
50-59 vs 60-69	6252146	.218643	5899969	.0000001*	5955613	.000003*
60-69 vs 70-79	3648908	.926536	3411866	.000044*	3554410	.089737
70-79 vs 80-89	113037.5	.044956	119751	.339229	120492	.397563

** Significance level is at p < .006*

Table 6. Mean Scores of THS subscales for the total UK Healthy sample and by gender. Min-max values are presented in parentheses and ± represent standard deviation (SD).

Group	Number of Participants	Engagement in Tactile Treatments	Communication facilitation via touch	Comfort with Touch in Medical Settings
Total sample	12,291	13.29 ± 3.70 (4-20)	14.13 ± 3.54 (5-25)	11.58 ± 2.17 (3-15)
Females	9,346	13.38 ± 3.79 (4-20)	13.87 ± 3.49 (5-25)	11.43 ± 2.16 (3-15)

Males	2,945	12.98 ± 3.34 (4-20)	14.97 ± 3.55 (5-25)	12.06 ± 2.14 (3-15)

Correlations of THS subscales with Psychological traits

Table 7. Pearson Correlation coefficients (r) between THS subscales and loneliness measured by the UCLA scale using of 4 and 8 items and interoceptive accuracy measured by the 16-item IAS scale.

Instrument		THS subscales		
		Engagement in Tactile Treatments	Communication Facilitation via Touch	Comfort with Touch in Medical settings
UCLA	4-item[a]	-.214*	-.093*	-.175*
	8-item[b]	-.179*	-.052*	-.153*
IAS	16-item[c]	.115*	.063*	.069*

**p < .001*
a: 4-item UCLA short version according to Russell et al., 1980.
b: 8-item UCLA short version according to Hays and DiMatteo, 1987.
c: 5 items related to breathing, cough, temperature, tired/sore muscles and taste were excluded due to their relation to COVID19.

Regression Analysis

Personality Traits

Table 8. Summary of the hierarchical regression analysis for personality traits predicting Communication Facilitation via Touch scores, r indicates zero order correlations.

Predictor Variables	B	SE(B)	Beta	t	Sig.	r
**Model 1**						
Age	.029	.002	.110	12.376	<.001	
Gender	-1.051	.074	-.127	-14.236	<.001	
Completion Date	-.004	.002	-.020	-2.203	.028	
**Model 2**						
Age	.025	.002	.097	10.975	<.001	
Gender	-1.319	.074	-.159	-17.873	<.001	
Completion Date	-.003	.002	-.018	-2.080	.038	
Extraversion	.156	.009	.174	17.972	<.001	.182*
Agreeableness	.106	.012	.080	9.015	<.001	.102*
Openness	.067	.011	.056	6.112	<.001	.122*
Neuroticism	.027	.008	.031	3.291	.001	-.072*

**p < .001*

Attachment style

Table 9. Summary of the hierarchical regression analysis for avoidant and anxious attachment styles predicting Communication Facilitation via Touch scores, r indicates zero order correlations.

Predictor Variables	B	SE(B)	Beta	t	Sig.	r
**Model 1**						
Age	.029	.002	.111	12.400	<.001	
Gender	-1.045	.074	-.126	-14.073	<.001	
Completion Date	-.004	.002	-.022	-2.439	.015	
**Model 2**						
Age	.036	.002	.138	15.502	<.001	
Gender	-1.064	.073	-.129	-14.629	<.001	
Completion Date	-.004	.002	-.019	-2.187	.029	
Avoidant	-.078	.004	-.162	-18.314	<.001	-.139*

Attachment						
Anxious Attachment	.056	.004	.132	14.798	<.001	.095*

**p < .001*

Body Acceptance

Table 10. Summary of hierarchical regression analysis for body acceptance predicting Engagement in Tactile Treatments scores, r indicates zero order correlations.

**Predictor Variables**	**B**	**SE(B)**	**Beta**	**t**	**Sig.**	**r**
**Model 1**						
Age	.011	.002	.041	4.460	<.001	
Gender	.403	.079	.047	5.111	<.001	
Completion Date	.000	.002	.002	.248	.804	
**Model 2**						
Age	.006	.002	.023	2.544	.011	
Gender	.569	.079	.066	7.246	<.001	
Completion Date	.000	.002	.001	.105	.917	
Body acceptance	.132	.008	.155	16.927	<.001	.149*

**p < .001*

Table 11. Summary of the hierarchical regression analysis for body acceptance predicting Communication Facilitation via Touch scores, r indicates zero order correlations.

**Predictor Variables**	**B**	**SE(B)**	**Beta**	**t**	**Sig.**	**r**
**Model 1**						
Age	.029	.002	.112	12.438	<.001	

Gender	-1.055	.074	-.127	-14.166	<.001	
Completion Date	-.004	.002	-.021	-2.333	.020	
**Model 2**						
Age	.026	.002	.102	11.264	<.001	
Gender	-.962	.075	-.116	-12.865	<.001	
Completion Date	-.004	.002	-.022	-2.428	.015	
Body acceptance	.074	.007	.090	9.976	<.001	.118*

*$p < .001$

Table 12. Summary of the hierarchical regression analysis for body acceptance predicting Comfort with Touch in Medical settings scores, r indicates zero order correlations.

**Predictor Variables**	**B**	**SE(B)**	**Beta**	**t**	**Sig.**	**r**
**Model 1**						
Age	.020	.001	.123	13.646	<.001	
Gender	-.603	.046	-.119	-13.198	<.001	
Completion Date	-.001	.001	-.007	-.741	.459	
**Model 2**						
Age	.016	.001	.102	11.489	<.001	
Gender	-.489	.045	-.096	-10.810	<.001	
Completion Date	-.001	.001	-.008	-.925	.355	
Body acceptance	.090	.004	.179	19.990	<.001	.204*

*$p < .001$

Interoceptive Accuracy

Table 13. Summary of the hierarchical regression analysis for interoceptive accuracy predicting Engagement in Tactile Treatments scores, r indicates zero order correlations.

**Predictor Variables**	**B**	**SE(B)**	**Beta**	**t**	**Sig.**	**r**

**Model 1**						
Age	.011	.002	.041	4.515	<.001	
Gender	.399	.079	.046	5.069	<.001	
Completion Date	.001	.002	.003	.294	.769	
**Model 2**						
Age	.009	.002	.032	3.540	<.001	
Gender	.327	.079	.038	4.161	<.001	
Completion Date	.001	.002	.006	.689	.491	
Interoceptive Accuracy	.038	.003	.109	11.966	<.001	.114*

**p < .001*

Table 14. Summary of the hierarchical regression analysis for interoceptive accuracy predicting Communication Facilitation via Touch scores, r indicates zero order correlations.

**Predictor Variables**	**B**	**SE(B)**	**Beta**	**t**	**Sig.**	**r**
**Model 1**						
Age	.029	.002	.112	12.399	<.001	
Gender	-1.055	.074	-.128	-14.187	<.001	
Completion Date	-.004	.002	-.021	-2.314	.021	
**Model 2**						
Age	.028	.002	.107	11.864	<.001	
Gender	-1.091	.074	-.132	-14.647	<.001	
Completion Date	-.004	.002	-.019	-2.112	.035	
Interoceptive Accuracy	.019	.003	.056	6.226	<.001	.055*

**p < .001*

Table 15. Summary of the hierarchical regression analysis for interoceptive accuracy predicting Comfort with Touch in Medical settings scores, r indicates zero order correlations.

Predictor Variables	B	SE(B)	Beta	t	Sig.	r
**Model 1**						
Age	.020	.001	.123	13.653	<.001	
Gender	-.603	.046	-.119	-13.199	<.001	
Completion Date	-.001	.001	-.006	-.695	.487	
**Model 2**						
Age	.019	.001	.117	13.001	<.001	
Gender	-.630	.046	-.124	-13.790	<.001	
Completion Date	.000	.001	-.004	-.442	.659	
Interoceptive Accuracy	.014	.002	.070	7.753	<.001	.070*

**p < .001*

Mental Wellbeing (SWEMWBS**)**

Table 16. Summary of the hierarchical regression analysis for THS subscales predicting Mental Wellbeing scores, r indicates zero order correlations.

Predictor Variables	B	SE(B)	Beta	t	Sig.	r
**Model 1**						
Age	.039	.003	.127	14.182	<.001	
Gender	.502	.088	.051	5.696	<.001	
Completion Date	-.007	.002	-.030	-3.391	.001	
**Model 2**						
Age	.036	.003	.117	13.120	<.001	
Gender	.475	.089	.048	5.333	<.001	
Completion Date	-.007	.002	-.031	-3.538	<.001	
EET	.139	.012	.123	11.384	<.001	.138*
CFT	-.084	.013	-.070	-6.584	<.001	.042*
CTM	.203	.020	.105	10.268	<.001	.137*

**p < .001*
ETT= Engagement in Tactile Treatments
CFT= Communication Facilitation via Touch

CTM= Comfort with Touch in Medical settings
